# The impact of testosterone therapy on quality of life in adolescents with Duchenne muscular dystrophy

**DOI:** 10.1016/j.nmd.2021.09.007

**Published:** 2021-12

**Authors:** CL Wood, J Page, J Foggin, M Guglieri, V Straub, TD Cheetham

**Affiliations:** a Translational and Clinical Research Institute, Newcastle University UK; b Department of Paediatric Endocrinology, Newcastle upon Tyne Hospitals NHS Foundation Trust UK; c John Walton Muscular Dystrophy Research Centre, Newcastle University and Newcastle Hospitals NHS Foundation Trust UK

**Keywords:** Duchenne muscular dystrophy: Puberty, Short stature, Quality of life, Testosterone

## Abstract

Duchenne muscular dystrophy (DMD) is the most common muscular dystrophy in childhood. It is associated with progressive muscle function decline and premature death. Long-term oral glucocorticoid use slows muscle weakness but is associated with several side effects including delayed puberty. This study assessed the impact of a 2-year incremental intramuscular testosterone regimen on quality of life (QoL) in a cohort of 15 adolescents with DMD. The Pediatric Quality of Life Inventory (PedsQL) Neuromuscular module was used to assess QoL and was completed by parent-child dyads. Semi-structured interviews were carried out to understand patient views on testosterone therapy. QoL scores increased in 10 of the 15 participants during treatment, with a mean total PedsQL score of 74.6 pre-treatment v 80.2 post treatment (*p* = 0.04). This was supported by comments in the semi-structured interviews. Parent-reported PedsQL scores were lower than their child's post treatment (*p* = 0.007). Testosterone therapy for pubertal induction was associated with an improvement in QoL and the observed physical changes during puberty played an important role. Low self-esteem was also a prevailing theme. This data supports the inclusion of testosterone therapy for pubertal induction as a Standard of Care.

## Highlights

• Testosterone therapy for pubertal induction was associated with improvements in QoL.
• Physical changes during puberty played an important role.
• Low self-esteem was also a prevailing theme.
• Parent-reported QoL scores were lower than patient-reports, consistent with other literature.
• Data from this study will provide a useful foundation for future work.

## 1 Introduction

Duchenne muscular dystrophy (DMD) is the most common muscular dystrophy of childhood and affects 1 in 5000 live male births [1]. There is no cure for DMD but therapeutic advances such as the routine use of glucocorticoids (GC) and coordinated multidisciplinary care have improved the course of the disease [2] so that patients regularly survive into their thirties [3]. Unfortunately the long term use of high-dose GC comes at a price and patients experience side-effects that can cause considerable morbidity. Of these, growth failure and pubertal delay are two of the main concerns reported by adolescents [4]. As the health of adolescents with DMD has improved, the expectations of the patients have also changed, with more seeking to establish relationships and to lead independent adult lives. Adverse psychosocial and educational consequences of failure to advance through puberty are well recognised in healthy individuals, but there is no qualitative work in the DMD population.

We recently completed a clinical trial where pubertal induction was carried out using a 2-year incremental regimen of intramuscular testosterone to determine its acceptability and effect on measures of satisfaction, pubertal staging, muscle function and quality of life. The quantitative outcome measures of this study have been reported elsewhere [5]; here we explored the psychological challenges faced by these adolescents and their views regarding the impact of testosterone supplementation on their quality of life.

The objectives were to use:

(1) The Pediatric Quality of Life questionnaire (PedsQL) to assess quality of life and
(2) Semi-structured interviews to gain a deeper understanding of the challenges faced in the adolescent with DMD and during pubertal induction.

## 2 Materials and methods

### 2.1 Participants

Participants were part of a single center study (NCT02571205) following the progress of 15 adolescents with DMD and delayed puberty as they were treated with testosterone to induce puberty. Males aged between 12 and 17 years old who were receiving GC were recruited between December 2015 and December 2016. Full details of the protocol have been published [6]. Briefly, patients were treated with a standard stepwise regimen of testosterone, using Sustanon [7] via intramuscular injection every 4 weeks for 2 years. Ethical approval was obtained from the York NHS Research Ethics Committee (15/NE/0332).

### 2.2 Pediatric quality of life inventory neuromuscular module (PedsQL)

The 25-item PedsQL 3.0 Neuromuscular Module is validated for the assessment of QoL in DMD [8,9]. The PedsQL encompasses 3 scales: 1) disease process and symptomatology (17 items), 2) patient's ability to communicate with healthcare providers and others about his/her illness (3 items) and 3) family resources (5 items). The scales are comprised of parallel patient self-report and parent proxy report formats. Items are scored using a Likert response scale; data are then transformed to a 0–100 scale (never ∼100, almost never ∼75, sometimes ∼50, often ∼25, and almost always ∼0) where higher scores indicate better QoL. Participants and their parent/carer completed the questionnaire at 6-month intervals.

### 2.3 Semi – structured interviews

Participants were given the option of consenting to a semi-structured interview to gain insight into the boys’ views on the challenges they face and the use of testosterone therapy. Interviews with the adolescents were carried out by the same member of the study team (CW). Some of the participants chose to have a parent present during the interview. A sample question list was used as a guide and drawn up after consultation with a qualitative data analysis expert with individual interviews guided by responses. Coding and thematic analysis was used and themes explored until saturation [10]. The interviews were read by CW and then anonymised and read by two further researchers (JP and JF) who also coded them to highlight recurrent ideologies. Patterns in the data were condensed and reduced to themes and these were re-reviewed in order to ensure they accurately described content.

### 2.4 Statistical analysis

Paired t-tests were used to compare QoL before and after testosterone treatment and unpaired t-tests to compare parents estimations of their son's perceived QoL compared with the self-reported scores.

## 3 Results

### 3.1 PedsQL

**Fig. 1:**
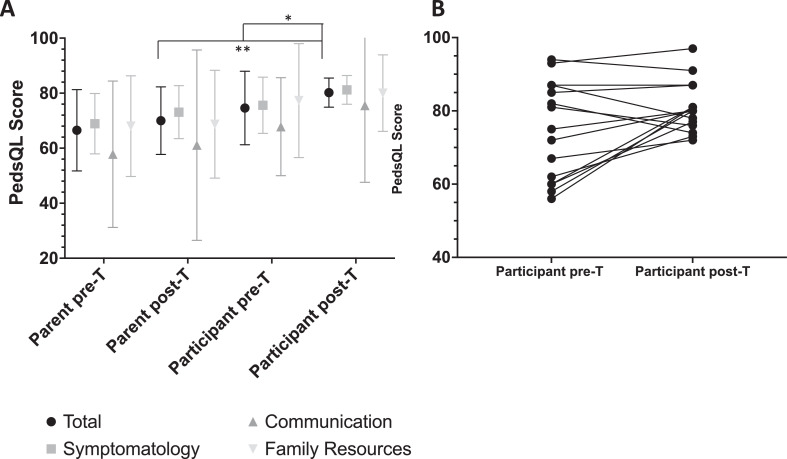
A Box and whisker plot (box- mean, whiskers- standard deviation) to show the patient and parent-reported mean total and category PedsQL scores before and after the incremental testosterone regimen. *indicates *P* = 0.04 for difference between patient total score pre and post-treatment, **indicates *P* = 0.007 for difference between patient and parent total score post-treatment. B shows the individual participant scores pre and post-T treatment.

PedsQL questionnaires were returned by participants and parents with no missing values. Two of the participants stopped treatment at 18 months, so their questionnaires from that timepoint were used as their final ones. Patient-reported mean PedsQL total scores were 74.6 (SD 13.4) out of a maximum of 100 at baseline. Following the 2-year incremental testosterone regimen, there was an increase to 80.9 (SD 6.9, *p* = 0.04) (table 1, Fig. 1). Mean scores also tended to be higher in each of the individual domains (disease/symptomatology, communication and family resources) post treatment. Ten of the 15 participants reported a higher PedsQL score following testosterone treatment. The increment by which patients reported an improvement in QoL (range 2–22, median 9.5) was higher than the reported decrease in QoL (range 3–9, median 5). Parent-reported PedsQL scores were similar pre- and post-treatment (66.5 (SD 14.8) v 70.0 (SD 12.3 *p* = 0.44)), although post-treatment mean scores were greater across all domains (Table 1, Fig. 1). The spread of scores in the communication domain was high (SD 26.6–34.6) indicating variability in parents’ perceptions of their child's ability to communicate about their disease with scores also tending to be lower than other domains (Table 1).

**Table 1:** PedsQL results *indicates *P* = 0.04 for difference between patient total score pre and post-treatment, **indicates *P* = 0.007 for difference between patient and parent total score post-treatment.

	Total score Mean (SD)	Disease/symptomatology Mean (SD)	Communication Mean (SD)	Family resources Mean (SD)
Patient pre-T	74.6 (13.4)	75.6 (15.0)	67.8 (17.8)	77.3 (20.7)
Patient post-T	80.9 (6.9)*	81.2 (8.4)	75.5 (27.9)	80.0 (13.9)
Parent pre-T	66.5 (14.8)	69.0 (15.5)	57.8 (26.6)	68.0 (18.3)
Parent post-T	70.0 (11.9)**	73.1 (14.1)	61.1 (34.5)	68.7 (19.6)

Patient-reported PedsQL scores were higher than parent-reported scores pre- (74.6 v 66.5; *p* = 0.12) and post-treatment (80.2 v 70.0; *p* = 0.007) (table 1). This suggests a tendency of family members to underestimate their child's quality of life with lower scores observed across all domains. This was most apparent in relation to patients’ ability to communicate about their illness (pre-treatment patient communication 67.8, parent 57.8 (*p* = 0.24) and post-treatment patient 75.5, parent 61.1 (*p* = 0.22)).

### 3.2 Semi-structured interviews

**Fig. 2:**
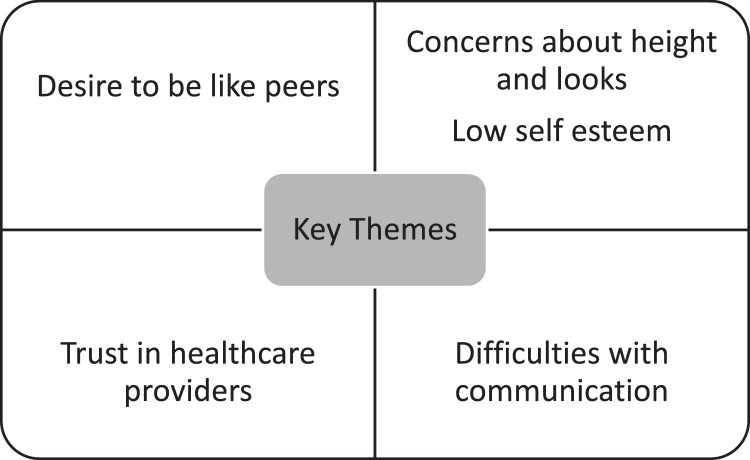
Key themes highlighted from the semi-structure interviews.

Ten out of the 15 participants agreed to be interviewed. The desire to be like their peers and a sense of *‘not fitting in’* was an overarching theme throughout the interviews (Fig. 2). Several of the boys commented on their inability to partake in activities like their friends, “*I try not to feel different but maybe I do, like if it's an activity I just can't do, I have to sit out.”* Most of the boys had begun noticing their peers developing faster than them:

“*I hate looking younger and shorte*r.”

“*I really don't like being small. I know people at school think I'm small.”* The main concern of the boys tended to relate to height and only one boy did not express this as a specific concern in his interview:

“*My size bothers me most- I've always felt small compared to the rest of my class”*

*“I'd like to carry on growing and be like my friends.”*

*“It's my height I would like to change.”*

Concerns regarding pubertal development were also expressed in a variety of ways including:

(a) Feeling self-conscious about the pitch of voice- “*I'm hoping my voice will change a bit”*
(b) Looking younger-*“Everyone when they look at me thinks that I am younger. They ask what year I am in, not in a nasty way, but am always surprised when I say….”*
(c) Lack of hair growth-*“Looking forward to being hairier.*”
(d) Penis size- *“I want to take part, to make going to the toilet easier, you know.”* (Mum then explained that toileting would be easier if his penis was bigger.)

If these individual concerns were grouped together to represent concern over ‘lack of pubertal development’ it may be considered as an equally common concern to short stature.

After testosterone treatment, several of the boys commented on feeling and looking older and having grown: *“The best thing to come from the testosterone injections is that I have grown taller.”*

*“I've grown in height and my voice has broken.”*

“*I feel more confident with young people of the same age group as myself.”*

*“I feel much better and stronger.”*

The theme of low self-esteem featured throughout the interviews (Fig. 2). Some boys expressed interest in pursuing more intimate relationships but did not have the confidence to do so. One boy described how he has thought about ‘asking a girl out’ but wasn't sure if he'd be able to, adding “*I would like a bit more confidence.”* Another boy described having to get changed separately from the other children at school for PE because he takes longer as “*a relief so people don't notice me”* and another said “*I don't like to use my wheelchair at school.”* Some parents (who sat in with their children at the request of the child) expressed concerns about confidence levels:

*“He definitely has low self-esteem.”*

*“He doesn't perceive that any girl would find him attractive. If he gets any attention, he's chuffed to bits.”*

Another theme was that of *difficulties negotiating transparency and openness,* referring to the young person's struggles to communicate with their peers about their condition (Fig. 2). Some of the boys said their closest friends were aware of their muscle condition but that it was not a topic of discussion. The boys communicated this in a way that suggested this was not out of malice but was likely an avoidance of difficult conversations; “*My best friend knows about it, but he doesn't really seem to notice, so we don't discuss it much*.” When another was asked what do your friends think about you having Duchenne? Do they ever talk about it with you, he replied *“Not my short term friends no. I try not to feel different from them.”*

*Trust in healthcare providers* describes the rapport built between participants and clinicians. Although at times there was uncertainty surrounding the delivery of testosterone via injection and growth failure as a side effect of their steroid medication, the boys said that their doctors were “*doing the right thing”.* Whilst several said that they would rather take an oral preparation, only one of the 7 patients expressed concern regarding having to inject testosterone. For example, when asked, “How do you feel about having injections:

*“I would rather take a tablet but if it needs to be an injection, that is fine too”*

*“An injection is a sharp pain, but it goes away quickly and if that is the best way to get the testosterone in, that's fine”.*

It was widely recognised among the boys that short stature was largely a result of their steroid medication but many acknowledged the beneficial effects of steroids in terms of preserving muscle function, *“I've thought about it a lot as I know they* (the steroids) *are making me short and look young but I am doing ok with them so don't want to stop.”*

## 4 Discussion

Higher PedsQL scores were reported by most participants at the end of the study, suggesting that the testosterone regimen was well-tolerated and that pubertal induction was associated with improved QoL. This is consistent with the TSQM data, which showed a mean total score of 48.5 (SD 6.2, range: 37–59) out of a possible 59 points, with similar scores in each domain. To the nearest integer, the mean score for all but one treatment domain was 6 (‘very satisfied’ [5]. Positive outcomes such as height gain and pubertal changes and improved confidence were reported by the boys in the semi-structured interviews. This, alongside the emotional maturation of the boys and therefore their ability to cope with the disease burden, are factors which likely contributed to the higher QoL scores following treatment. A study of subcutaneous testosterone therapy in adolescents with primary hypogonadism also demonstrated a clear improvement in psychosocial outlook and self-image [11]. This is consistent with reports of increased cheerfulness and relaxation in hypogonadal men receiving androgen [12,13]. In our experience, families can be concerned about potential negative effects of exogenous androgen on mood and behavior during adolescence but this was not raised as a significant issue by families during the course of the trial.

The most common concern amongst the study participants was short stature. Short stature combined with pubertal delay and therefore a lack of an associated pubertal growth spurt, facial hair and deepening of the voice often results in DMD boys being perceived to be much younger than their actual age, with one boy explaining “*in restaurants they never ask me what I'd like to eat, they always ask mum* [4].*”* Physical appearance has been identified as the single most important factor contributing to self-worth in adolescents, therefore the improvement in the majority of QoL scores may reflect change in appearance [14]. Severe short stature, such as that often observed in DMD, can be associated with emotional distress and may contribute to diminished QoL [15]. Other common concerns were having a high pitched voice and mobility difficulties, for example when changing clothes. The pubertal growth spurt results from increased sex hormone concentrations and growth hormone secretion [16]. Although ongoing glucocorticoid use will suppress the pubertal growth spurt, each participant experienced an increase in growth velocity during the study period.

It is acknowledged that it is difficult for adolescents to know how best to approach topics with sensitivity and this may inadvertently result in a lack of peer support. None of the boys reported talking to anyone else with DMD or using internet forums to meet other affected individuals but they still expressed an interest in doing so. The boys’ parents appear to be their main source of information with many saying that if they had any queries about their condition; “*I'd speak to mum or dad I suppose”.* In-depth interviews in another study of 7 boys with DMD also identified a reluctance to discuss problems with their families [17]. Our knowledge of the expectations of adults with DMD remains sparse and those studies that have been carried out show conflicting results. One study showed that, despite their physical limitations, adults with DMD perceive a high quality of life [18]. One study suggested that the ‘average adult with DMD had an outspoken need for love from a girlfriend’ and concluded that parents and professionals must ‘anticipate in all measures taken that the DMD boy grows up to manhood and will need competences for adult social life in all respects’ [19]. Another found that 30% of patients in their adult DMD clinic were receiving treatment for depression and that emotional, social and psychosocial well-being were significantly lower in adolescents with DMD [3]. Contrary to this, however, a large scale questionnaire study conducted in cooperation with the Duchenne Parent Project reported that only 17% of the patients had a low score for psychosocial adjustment – a measure of how one adapts to difficult and stressful events associated with disease. It is known that as boys with DMD grow older, their psychosocial adjustment score increases which means that they become better equipped to cope with disability [20] This may be partly reflected in the higher post-treatment scores and contrary to popular public perception, may also be related to loss of ambulation. Whilst this is often perceived as a devastating milestone, it can reduce fatigue and give the patient a greater feeling of independence.

A diagnosis of DMD can have a significant psychological impact on the patient, family and other caregivers [21]. The relentless, arduous disease course can lead to parental strains and unsurprisingly, mood disorders and disability are more prevalent in DMD families [22]. For many parents of children with DMD, the stress caused by psychosocial aspects of the child exceeds the stress caused by the physical aspects [23]. Parents seem to underestimate their children's QoL [24], [25], [26], [27], [28] which can be ascribed to disparities in perception of QoL. A consistent trend of underestimation of QoL by parents was observed in this study. Parents and caregivers are likely to adjust their perception of patients QoL based upon the disease trajectory [28]. It would appear that parents’ own worries and fears about their child's condition has an impact upon their assessment of their child's QoL and this is reflected in the disparities between QoL scores [29].

Poor confidence levels and low self-esteem are common traits of patients with chronic diseases such as DMD and this data supports this [21]. Some of the lowest levels of self-esteem in children with chronic illness have been reported in relation to fatigability, which is also a feature of DMD [30,31]. The reasons underpinning this low self-esteem are likely multidimensional and highly variable between conditions, but factors such as emotional support from parents and peers are important. There are a number of barriers which restrict social interaction and participation in DMD and it is probable that this negatively influences identity and self-concept. Participation in the study may have contributed to improved QoL [32]. Physical appearance is the single most important factor contributing to self-worth in adolescents with lower self-esteem observed in diseases where the disability is more visible [30]. Many reported positive changes as a result of the intervention, which may be one of the more important contributing factors to the higher reported PedsQL scores post testosterone treatment and is consistent with published data showing that testosterone levels correlate with confidence, anxiety levels and quality of life [33].

‘Difficulties negotiating openness and transparency’ was another theme isolated from the interviews and represents the inadvertent marginalization of these boys. This likely adds to poor self-image given that peer approval has been identified another leading source of children's self-esteem [30].

### 4.1 Study limitations

DMD is rare and the sample size was based on the available local population. This study consisted of 15 participants and hence is underpowered to detect small differences in PedsQL domains. Despite this, clear trends were visible, with higher reported PedsQL scores post treatment by both patients and parents. Increments in the range of 1–2 in the PedsQL scores are considered to be relatively insignificant changes to QoL scores and are likely reflective of natural fluctuations in mood rather than actual change in QoL [34]. An individual's mood on the day the questionnaire was administered is potentially a confounding factor. It is difficult to be able to define at which point the magnitude of the increase/decrease in PedsQL score becomes clinically significant. The 5 patients who reported diminished QoL scores did not have accompanying low TSQM scores [5] and no significant disease milestones or functional decline to set them apart from patients who reported improved QoL, suggesting that natural mood fluctuations may account for some of the variation in QoL scores. Another limitation was the absence of a control group which makes it more difficult to evaluate the effect of testosterone therapy. Due to the nature of treatment, it would be impossible to blind subjects to treatment and under guidance from the Research Ethics Committee, it was deemed unethical to withhold treatment [5].

## 5 Conclusions

Testosterone therapy was associated with improved QoL in adolescents with DMD. It appears that the physical changes and height gain that testosterone therapy induced played an important role in this regard. Low self-esteem was a prevailing theme in the adolescents and we found, like other researchers, that parents tend to underestimate their children's QoL.

This work adds to the evidence from our published data which suggest a favourable impact of testosterone on bone density, muscle morphology and muscle function in adolescents with DMD [5]. We advocate the routine and timely use of exogenous androgen to initiate puberty in boys with DMD who are on GC, as recommended in the revised DMD standards of care [35].

## Funding

This work was supported by Duchenne Now and the Medical Research Council/MDUK (MR/N020588/1).

## Conflict of Interest

None.
